# How relevant for you is to be a moral person? Polish validation of the Self-Importance of Moral Identity Scale

**DOI:** 10.1371/journal.pone.0255386

**Published:** 2021-08-03

**Authors:** Mariola Paruzel-Czachura, Mateusz Blukacz

**Affiliations:** Institute of Psychology, Faculty of Social Sciences, University of Silesia in Katowice, Katowice, Poland; Universitat de Valencia, SPAIN

## Abstract

**Objective:**

The Self-Importance of Moral Identity Scale (SMIS) was developed by Aquino and Reeds with the purpose of measuring how people evaluate their private (*Internalization* subscale) and public (*Symbolization* subscale) moral identity. SMIS has become commonly and broadly used in many studies. The aim of this paper is to validate the Polish version of SMIS by analyzing its structure and relation to similar measures (such as The Moral Self-Concept Scale developed by Stake and The Moral Self-Image Scale created by Jordan, Leliveld and Tenbrunsel), declared past prosocial behaviors and readiness to donate money.

**Methods:**

The translation-back-translation procedure was used to maintain semantic, idiomatic, and conceptual equivalence of the original scale. Throughout four separate studies the psychometric properties of the Polish version of the scale were assessed: Study 1 (*N* = 529) was carried out to derive the factor structure using the exploratory factor analysis (EFA) and cross-validate it; Study 2 (*N* = 602) and Study 3 (*N* = 899) were performed to confirm and replicate the structure with the confirmatory factor analysis (CFA); gender-balanced Study 4 (*N* = 862) was conducted to assess measurement invariance over gender using multigroup CFA, and to normalize the scale. Validity of the scale was assessed based on each study.

**Results:**

A stable two-factor structure using 10 items was replicated in four different samples. The results showed that reliability (α) was between 0.71 and 0.81 for *Internalization*, and 0.76 and 0.81 for *Symbolization*. Validity was confirmed in terms of the expected pattern of correlations with morality measures and factorial structure. Metric invariance across gender was confirmed with possible exception of factor loadings on two items regarding communication of values. Polish normalization for men and women was constructed.

**Conclusions:**

Polish validation of SMIS proved to be a structurally consistent and valid measure.

## Introduction

Psychology is affected by the replication crisis [1], which can be caused by many sources, including poor methodology of the original studies or the development of statistics (possibilities of more accurate measurement of variables). Additionally, previous studies focused on WEIRD samples [2]. For many years, the following opinion has been repeated at psychological lectures worldwide: “Psychological knowledge is based on American student samples and therefore we are not certain whether that knowledge is also true for other people in different parts of the world”. To solve this problem, psychologists started to study more extensively non-WEIRD samples and tried to replicate original findings. Furthermore, they also paid more attention to scale validation, including multiple studies on samples from different cultures or countries.

Moral psychology has been one of the most intensively developing areas in the recent years [3–6]. However, researchers still use the scales or questionnaires that were developed several decades ago, which are often based on gender-imbalanced or student-only samples. The scales are rarely re-evaluated and are still broadly used in various cultures and contexts. In the midst of replication crisis, it should also be borne in mind that psychometric properties and validity evidence also need to be replicable to prove construct equivalence across cultures [7]. It is relevant to verify whether popular scales are robust over time [8]. Moreover, as culture is also relevant in moral psychology studies [9, 10], the scales should be validated in cultures they are used in. The aim of our study was to challenge this methodological issue and validate the Polish version of one of the most popular scales used in moral psychology, i.e., the Self-Importance of Moral Identity Scale (SMIS) developed by Aquino and Reed in 2002 [11]. We conducted a series of four studies to verify the structure and validity of the scale in the Polish population.

## Moral identity: *Internalization* and *Symbolization*

The concept of moral identity is well established in psychology and many studies have been conducted in this area [12, 13]. According to the classical definition of moral identity, it is a self-conception organized around a set of moral traits, which is relatively stable over time, but it is not a personality characteristic [11]. It means that people think about themselves as having some moral traits (e.g., being honest or fair). At the same time, their moral identity has a social referent such as a membership in some prosocial group, being a volunteer, etc. Therefore, moral identity has two dimensions of self-importance, i.e., private, and public. The *Internalization* dimension is related to the self-importance of moral characteristics, whereas *Symbolization* dimension is connected with a more general sensitivity to the moral self as a social object whose behaviors can demonstrate that one has these characteristics [11, 14]. The former dimension describes how much relevant to people is to have moral traits, while the latter describes how much relevant to them is to show others that they have moral traits.

In the original series of studies [11], both dimensions predicted the emergence of a moral spontaneous self-concept, and self-reported volunteering, but only the *Internalization* dimension predicted actual donation behavior. The *Internalization* dimension showed the strongest relationships to normlessness and moral reasoning, whereas the *Symbolization* dimension was more strongly correlated with religiosity. *Internalization* was more strongly related to the implicit measure that assesses the strength of the association between the moral traits and the self-concept.

It is understood that people with a strong moral identity should strive to maintain consistency between their moral self and their behaviors [15, 16]. It has been proven that SMIS is related to actual moral behaviors. Research has demonstrated that moral identity is related to prosocial/moral behaviors such as self-reported volunteerism and actual donation behavior [11, 13, 17]. The relationship was later confirmed in a meta-analysis based on more than 100 studies from a broad range of academic fields, including business, developmental psychology, education, marketing, sociology, and sport sciences [18].

## The Self-Importance of Moral Identity Scale

Almost 20 years ago Aquino and Reed [11] aimed to create a measure of how people evaluate their private (*Internalization* subscale) and public (*Symbolization* subscale) moral identity. First, in two pilot studies (*N* = 228; *N* = 137), they identified a set of moral traits that could reliably invoke moral identity. Then, in Study 1 (*N* = 363), the underlying factor structure of the new items was examined using exploratory factor analysis (EFA). Some items were adopted from previous instruments and some were formed based on the literature. Two factors, *Internalization* and *Symbolization*, with acceptable *α* reliability of .71 and .77 were found. This structure was confirmed using confirmatory factor analysis (CFA) on a new sample (*N* = 347). The correlated between scales was *r* = .44 (*p* = .001), with *α* of .73 and .82 for the *Internalization* and *Symbolization* scales, respectively. Next, examination of the convergent, nomological, and discriminant validity of the revised explicit measure was warranted to provide additional evidence of construct validity of five samples (total *N* = 324). The results supported the construct validity of the 10-item measure of the self-importance of moral identity.

SMIS has been used in many fields of psychology and related disciplines, especially in business studies and has been cited more than 2800 times. The scale has been applied in many studies, however, mainly in American and Canadian cultures. Validations of the scale have been published in other languages, including Iranian [19] and Czech [20], but they were done on small student samples. Also, some studies were conducted in non-English countries such as Spain. However, authors reported *α* reliability of .70 [21]. It is worth noticing that sometimes a short name for the scale is used, i.e., “moral identity scale”.

## Overview of the paper

The aim of the paper was to evaluate the psychometric properties and external validation of SMIS for the Polish population across a series of online studies. Although each study had its own objective, they all included SMIS and constructs that served as evidence of validity such as the Moral Self-Concept (MSC), Moral Self-Image (MSI), past prosocial behaviors (PPB) and declared readiness to donate money (RDM).

Studies were reported in chronological order to reflect the process of constructing and validating the Polish version of SMIS. Each subsequent study was used to provide more validation evidence and to resolve doubt and limitations found in previous studies. Study 1 was used to explore and cross-validate the factorial structure of SMIS; Study 2 served as a confirmation sample for that structure; Study 3 was used to assess replicability of the findings on general population and Study 4 yielded a representative gender-balanced sample to normalize the scale. Gender measurement invariance was tested on all four samples. In total, 2892 participants were enrolled in the analyses.

The studies were approved by the Ethical Committee of the University of Silesia in Katowice (Studies 1, 2, 3; number 1/2019) and the Ethical Committee of the Nicolaus Copernicus University in Torun (Study 4; number 1/2020/FT). The datafiles are available at OSF: https://osf.io/mkxay/.

## Study 1. Factorial structure assessment

The aim of the study was to explore the relationships between unethical behaviors, pornography, and beliefs about one’s morality.

## Materials and methods

### Measures

#### Moral identity

It is an identity construct that provides information on the extent to which a person values moral traits, and whether one’s actions demonstrate a commitment to moral self-expression. It was measured by SMIS [11], which is comprised of two dimensions, with 5 questions each. The *Internalization* subscale addresses the extent to which a person values moral traits (e.g., “I strongly desire to have these characteristics”), and the *Symbolization* subscale seeks to identify whether one’s actions demonstrate a commitment to moral self-expression (e.g., “The fact that I have these characteristics is communicated to others by my membership in certain organizations”). Participants are asked to imagine how a person with given traits (caring, compassionate, fair, friendly, generous, helpful, hardworking, honest, and kind) would feel, act, and think. Next, participants are asked to answer 10 questions on a 5-point scale from 1 (*strongly disagree*) to 5 (*strongly agree*). SMIS was translated into Polish (see supporting information) using translation-backtranslation [22, 23] to achieve accurate translation and maintain correspondence to the actual cultural context. Four researchers-translators (including two non-academics) worked on the scale under the supervision of the language coordinator. The first Polish version of the scale was translated independently by two translators who then discussed the final version A. The next step was back translation of the Polish version into English. Two other researchers independently translated version A back into English and then discussed the versions to create version B. Next, the language coordinator switched versions A and B between the pairs of translators whose comments were then used to form version C in Polish. The final step was the external evaluation of version C by two non-academics who were then interviewed on how they perceived and understood the translation. Their comments were noted, discussed, and implemented into the final Polish version (D). The procedure allowed the detection of items that might have culturally different meaning. For example, ‘books and magazines’ in item 8 were translated into ‘books and articles’ since the Internet and digital media had exceeded the number of printed magazines in the recent decade.

#### Moral self-concept

It is a subjective self-evaluation of the degree to which a person has moral traits. It was measured by the Moral Self-Concept Scale (MSCS) [24]. It is a subscale of the Six-Factor-Self-Concept Scale. In MSCS people indicate how accurately six adjectives (loyal, truthful, law-abiding, faithful, trustworthy, and honest) describe them on a 7-point scale from 1 (*never or almost never true of me*) to 7 (*always or almost always true of me*).

#### Moral self-image

It is a person’s dynamic and malleable moral self-concept. It provides the insight into moral self-perceptions, but unlike moral identity, MSI is assumed to be susceptible to events with a moral component and thus it is not expected to be stable over time [25]. The Moral Self-Image Scale (MSIS) was used to measure this variable [25]. The scale presents nine items consisting of the same traits as in the case of SMIS perceived as prototypical of the ideally moral person. The participants were asked to indicate their relative position to their own ideal self on each trait (e.g., for care: 1 –*much less than the caring person I want to be*; 9 –*much more than the caring person I want to be*).

#### Past prosocial behaviors

This construct represents prosocial real-life activities of a person. We used behaviors following Ray’s [26] views on high social consensus and asked the participants to respond how often they had (1) donated money to charity, (2) donated time as a volunteer, (3) donated blood, or (4) other prosocial behaviors (not specified) in the past year using a 6-point scale ranging from 0 (*never*) to 5 (*many times*). These items were averaged into an aggregate score on Past Prosocial Behaviors (PPB) as in the case of Vecina and Marzana [21].

#### Readiness to Donate Money (RDM)

The participants were asked what they would do if they had additional money (equivalent of $25) whether they kept it to themselves or donated some of it for prevention of pornography (as the use of pornography was the focus of this study). The answer to donate “nothing” was coded as 0, and the answers to donate any amount of money were coded as 1. This was treated as an ad hoc measure for RDM.

Apart from these measures, the participants were asked about their own unethical behaviors related to pornography. The questions regarding unethical behaviors were asked at the end of the survey not to affect the answers related to the moral self-evaluations of the participants. As this was beyond the scope of this paper, neither those questions nor the results were included.

### Participants and procedure

Five hundred and twenty-nine individuals (*N* = 529; 81% women) aged 18 to 54 years (*M* = 23.69, *SD* = 5.54) participated in the study and completed the whole survey. Students accounted for 49% (*n* = 258) of the sample, 30% (*n* = 160) of participants had higher education, 20% (*n* = 104) secondary education (or secondary with additional course/s), and the rest of the sample had primary education (1%; *n* = 7). Of note, 49% (*n* = 259) of the sample considered themselves religious (question: “Are you a religious person?”). However, 70% (*n* = 370) declared themselves as Catholics. All participants were informed about the aim of study and gave their informed consent. The participants were not given monetary compensation. We used convenience sampling and the snowball technique via social media (particularly Facebook). The descriptive statistics of psychological measures (SMIS, MSCS, MSIS, PPB and RDM) are given in Table 1.

**Table 1:** Means and standard deviations of SMIS, MSCS, MSIS, PPB and RDM in Studies 1, 2, 3 and 4.

		*Study 1*		*Study 2*	*Study 3*	*Study 4*		
		Subsample A	Subsample B	*N* = 602	*N* = 899	*N* = 862	male	female
		*n* = 264	*n* = 265				*n* = 435	*n* = 426
Age		23.65 (5.49)	23.74 (5.59)	22.13 (2.14)	32.23 (13.02)	49.31 (16.51)	51.07 (16.30)	47.51 (16.55)
*Moral Identity*	item 1	4.16 (0.88)	4.27 (0.79)	4.33 (0.80)	4.32 (0.83)	4.29 (0.76)	4.19 (0.73)	4.39 (0.78)
	item 2	3.94 (0.86)	4.04 (0.83)	4.04 (0.90)	4.07 (0.87)	4.15 (0.78)	4.01 (0.76)	4.28 (0.78)
	item 3	4.28 (0.80)	4.33 (0.79)	4.26 (0.98)	4.32 (0.95)	4.52 (0.88)	4.43 (0.92)	4.61 (0.83)
	item 4	3.84 (1.10)	3.87 (1.14)	3.75 (1.22)	4.09 (1.12)	3.74 (1.28)	3.63 (1.23)	3.85 (1.33)
	item 5	3.71 (0.97)	3.76 (0.95)	3.77 (1.07)	3.92 (1.01)	3.84 (0.95)	3.73 (0.95)	3.96 (0.94)
	item 6	2.70 (0.88)	2.68 (0.92)	2.55 (1.14)	2.59 (1.07)	2.76 (1.06)	2.60 (1.03)	2.92 (1.07)
	item 7	3.20 (1.01)	3.22 (1.01)	3.03 (1.09)	3.14 (1.05)	3.45 (0.90)	3.30 (0.92)	3.59 (0.87)
	item 8	2.93 (0.97)	2.97 (1.02)	2.83 (1.15)	3.07 (1.11)	3.25 (1.02)	3.08 (1.03)	3.42 (0.99)
	item 9	2.81 (1.24)	2.74 (1.16)	2.53 (1.27)	2.71 (1.22)	2.76 (1.15)	2.68 (1.15)	2.84 (1.15)
	item 10	3.20 (1.12)	3.17 (1.11)	2.87 (1.23)	3.17 (1.18)	2.99 (1.03)	2.88 (1.02)	3.11 (1.02)
	*Internalization*	3.99 (0.68)	4.06 (0.63)	4.03 (0.72)	4.15 (0.72)	4.11 (0.65)	4.00 (0.64)	4.22 (0.64)
	*Symbolization*	2.97 (0.80)	2.96 (0.78)	2.76 (0.89)	2.93 (0.83)	3.04 (0.74)	2.91 (0.74)	3.18 (0.72)
*Moral Self-Concept*		5.95 (0.67)	6.02 (0.68)	5.56 (0.77)	5.89 (0.68)			
*Moral Self-Image*		5.32 (1.33)	5.51 (1.33)	4.48 (1.10)	4.86 (1.09)			
*Past Prosocial Behaviors*		3.02 (1.06)	2.94 (0.98)	1.75 (0.97)				
*Readiness to Donate Money (n*, *%)*		115 (44%)	124 (47%)	174 (29%)	167 (85%)	759 (88%)	368 (85%)	391 (92%)

## Study 2. Confirmation of the factorial structure

Study 2 was related to unethical online behaviors, including illegal downloading of software and files. As in the case of Study 1, the questions about unethical behaviors were asked at the end of the survey. Due to some concerns about Study 1, this study was intended to provide a more-gender balanced and homogeneous sample.

### Measures

The same measures as in Study 1 were used. In the RDM measure, the participants were asked if they would donate money ($25) for prevention of illegal downloading. The participants also answered questions regarding their unethical behaviors related to illegal downloading. These results, however, are not reported as they are not within the scope of this paper.

### Participants and procedure

A group of 602 university students (62% women, *n* = 374) participated in a voluntary online survey based on a convenient sampling method without monetary compensation. The participants aged 18 to 30 with the mean age of 22.13 (*SD* = 2.14). 51% (*n* = 308) of the sample considered themselves religious and 68% (*n* = 412) declared themselves to be Catholic. The participants were informed about the aim of the study and gave their consent for participation. The descriptive statistics of psychological measures (SMIS, MSCS, MSIS, PPB and RDM) are given in Table 1.

## Study 3. Replicability of the factorial structure

Study 3 was related to unethical everyday life behaviors. The questions about unethical behaviors were asked at the end of every online survey to avoid potential influence of the answers on subjective moral self-evaluations of the participants (the project website is www.mojamoralnosc.pl/en; the study is available on the Polish version of the website).

In this study, the rationale for the use of SMIS was to validate it on general non-academic population bearing in mind that it might be a potential limitation of Studies 1 and 2.

### Measures

Apart from measuring PPB, the same measures were used as in Studies 1 and 2. In the RDM measure, the participants saw the same instruction and scale as in previous studies but here subjects were asked whether they would donate money (equivalent of $25) for prevention of the most unethical behavior which they had done in their lives (they did not have to specify it). The complete list of questions is available on the project website.

### Participants and procedure

In Study 3, 899 participants (76% women, *n* = 680) aged 18 to 80 (mean 32.23; *SD* = 13.02) participated in an online survey based on a convenient sampling method. 477 subjects (53%) had higher education, 381 (42%) secondary education or secondary with additional course(s) and 41 (5%) primary education. All participants were informed about the aim of the study and gave their informed consent for participation. The study was popularized in the media, including university and social media, radio talks about the project, and science festivals. No monetary compensation was given. However, the participants were provided with feedback with anonymous interpretation of their scores (after filling the survey, the system automatically counted the participants’ scores and the feedback appeared on the screen for personal use). The descriptive statistics of psychological measures (SMIS, MSCS, MSIS, PPB and RDM) are given in Table 1.

## Study 4. Gender invariance and normalization

Study 4 was an international collaboration on psychological, social, and moral aspects of Coronavirus Disease 2019 (COVID-19; the project website: https://icsmp-covid19.netlify.app/). In this paper, we analyzed the Polish data only. The sample was used for the validation of SMIS and normalization as it was gender-balanced and representative in terms of education and age which might not have been sufficient in Studies 1, 2 or 3.

### Measures

The Self-Importance of Moral Identity Scale and the modified measure for RDM were used. The participants were asked how much they would donate for charity organizations that worked full-time to protect people from COVID-19 if they were given the average daily wage in their country (equivalent of $50). We coded the answers to donate any amount of money as 1, otherwise 0. Apart from these measures, the participants were asked questions about their moral and social beliefs and COVID-19-related behaviors which were outside the scope of this paper. The complete information about the survey is available on the project website.

### Participants and procedure

Study 4 was conducted among 862 participants (50% women) aged 18 to 85 years (mean 49.31; *SD* = 16.51). The participants were recruited by research company independent of the authors (Pollster Research Institute) using online research platform which consist of 176 000 Poles. Quota sampling according to demographic characteristics (education, age, and gender) was used to reflect the structure of Polish population in accordance with the data published by Statistics Poland (https://stat.gov.pl/en/).

69 participants (8%) had primary education, 238 (28%) vocational education, 318 (37%) high school education and 237 (27%) higher education. 396 participants (46%) were employed full time, part time, or were self-employed, 267 (31%) were retired, 87 (10%) were unemployed, and 114 (13%) were students or had other employment status. All participants were informed about the aim of the study and gave their informed consent for participation. The subjects received small monetary compensation. The descriptive statistics of psychological measures (SMIS, MSCS, MSIS, PPB and RDM) are given in Table 1.

### Data analysis

Study 1 was used to assess factorial structure of the construct using the analytic strategy similar to the one applied by Aquino & Reed [11]. The sample was split into two equal-sized subsamples (A and B). Parallel analysis (PA) [27, 28] and exploratory graph analysis (EGA) using graphical LASSO estimation and *walktrap* algorithm [29, 30] were used to establish dimensionality, which was followed by EFA with oblique and orthogonal rotations with primary factor extraction to derive the factor structure. In the second step, CFA with maximum likelihood (ML) estimation was used to verify that structure on subsample B. CFA models were adjusted by adding covariances between pairs of items based on similarity of their contents and values of modification indices (MI) to achieve satisfactory fit. Model fit was assessed using the Comparative Fit Index (CFI) [31], Tucker-Lewis Index (TLI) [32], Root Mean Square Error of Approximation (RMSEA) with 90% CI [33, 34], the Akaike Information Criterion (AIC) and the Bayesian information criterion (BIC). A good fit was recognized when CFI and TLI were higher than 0.90 and RMSEA was close to 0.08 [35] and an excellent fit when CFI and TLI were higher than 0.95 and RMSEA was lower than 0.07 [36]. Since Aquino & Reed [11] used Root Mean Square Residual (RMSR) we also reported it for comparison. The models were compared using the Likelihood Ratio (LR) test. The convergent validity evidence for distinguished factors was assessed by examining their relations to other morality constructs, MSC, MSI, PPB and RDM, using Pearson’s correlations, partial correlations, logistic regression and *t*-tests. Odds ratio (OR) were used to quantify relation of odds of donating money and SMIS point scores. Reliability was assessed using the alpha coefficient [37].

Study 2 was used to confirm the factor structure with CFA and assess validity on more gender-balanced sample. Study 3 was planned to test the replicability of the factor structure with CFA and validity evidence from Study 1 and 2. Study 4 was used to evaluate the consistency of the factor structure with CFA on an independently collected sample. Studies 1–4 were used to test measurement invariance between men and women with multigroup CFA. Both factor structure and measurement invariance were interpreted in terms of validity evidence regarding internal structure. Study 4 was also used to create Polish normalization of SMIS (see S1 Table). Analyses were performed using STATA/SE 14.2 [38] and R package *EGA* [30].

## Results

### Study 1

Dimensionality analysis and EFA of SMIS items were conducted on subsample A of 264 participants (80% women) form Study 1. Parallel Analysis (PA) revealed four empirical eigenvalues higher than the random generated eigenvalues, and eigenvalues plot suggested existence of two major dimensions and probable two minor dimensions. Exploratory graph analysis (EGA) was used to further explore dimensionality resulting in detection of two main dimensions containing items 1–5 and items 6–10 respectively. Based on both analyses we determined existence of two factors. The eigenvalues PA plot and EGA graph are given in S1 and S2 Figs.

Next, Varimax and Promax (*κ* = 3) rotations were used, both resulting in two-factor structure with the first factor loading items 1–5 and second items 6–10. Aquino and Reed [11] used Varimax but due to a substantive correlation between factors (*r*_*F1F2*_ = 0.43) Promax rotation was more adequate to our data. Factor loadings led to two-factor solution (Table 2). Based on item loadings, factors of *Internalization* and *Symbolization* were identified (with factor loadings *λ*_*1–5*_ between 0.55 and 0.74 on items 1–5, and *λ*_*6–10*_ between 0.58 and 0.75 on items 6–10, respectively). Reliability was assessed using the alpha coefficient and was rather high (*α*_*Internalization*_ = 0.78 and *α*_*Symbolization*_ = 0.81).

**Table 2:** Exploratory Factor Analysis (EFA) factor loadings of SMIS items.

*Item*	* *	*Symbolization*	*Internalization*
1	It would make me feel good to be a person who has these characteristics.	-0.09	**0.67**
2	Being someone who has these characteristics is an important part of who I am.	0.22	**0.62**
3	I would be ashamed to be a person who has these characteristics. (R)	-0.10	**0.55**
4	Having these characteristics is not really important to me. (R)	0.05	**0.59**
5	I strongly desire to have these characteristics.	0.02	**0.74**
6	I often wear clothes that identify me as having these characteristics.	**0.58**	0.09
7	The types of things I do in my spare time (e.g., hobbies) clearly identify me as having these characteristics.	**0.75**	-0.02
8	The kinds of books and magazines that I read identify me as having these characteristics.	**0.58**	0.07
9	The fact that I have these characteristics is communicated to others by my membership in certain organizations.	**0.73**	-0.06
10	I am actively involved in activities that communicate to others that I have these characteristics.	**0.72**	0.08
*Eigenvalue*		2.87	2.62
*% variance explained*		68%	62%

*Note*: Items had a 5-point Likert scale (1—*strongly disagree*, 5—*strongly agree*); R—reverse-coded items; % variance explained sum is >100% due to correlated factors.

Subsample B (*n* = 265) was used to verify the factor structure of SMIS items from subsample A with CFA. The two-factor model with a simple structure (i.e., items load one factor with no covariances between items or secondary loadings on other factors [39]) was tested and proved nearly excellent fit (Model 1: RMSEA = 0.06, 90%CI 0.04–0.08, CFI = 0.95, TLI = 0.94, AIC = 6664.84, BIC = 6775.81). Possible fit improvements were explored by analyzing item contents and modification indices (MI). We decided to add covariance of items 9 and 10, as they both refer to communicating moral characteristics to other people, which significantly improved model fit (LR test *χ*^2^(1) = 27.41 *p*<0.001, Model 2: RMSEA = 0.03, 90%CI 0.00–0.06, AIC = 6639.42, BIC = 6753.98, CFI = 0.99, TLI = 0.96). To further improve fit, we allowed covariance of items 3 and 4, as they are the only items negating having moral characteristics (reverse coded items), which resulted in better fitting model (LR test *χ*^2^(1) = 6.09, *p* = 0.014, Model 3: RMSEA = 0.02, 90%CI 0.00–0.05, AIC = 6635.33, BIC = 6753.47, CFI = 1.00, TLI = 1.00). No basis for covariances between other items regarding their contents were found. CFA model is given in Table 3. The reliability of measures in subsample B was *α*_*Internalization*_ = 0.74 and *α*_*Symbolization*_ = 0.80, *α*_*MSCS*_ = 0.72, *α*_*MSIS*_ = 0.85 and *α*_*RDM*_ = 0.63.

**Table 3:** Factor loadings and model fit indices of Confirmatory Factor Analysis (CFA) of SMIS items in Studies 1, 2, 3 and 4.

		Study 1 (Subsample B, *n* = 265)			Study 2 (*n* = 602)			Study 3 (*n* = 899)			Study 4 (*n* = 862)		
		Model 1	Model 2	Model 3	Model 1	Model 2	Model 3	Model 1	Model 2	Model 3	Model 1	Model 2	Model 3
		*b*	*b*	*b*	*b*	*b*	*b*	*b*	*b*	*b*	*b*	*b*	*b*
*Internalization*	item 1	0.70	0.70	0.70	0.76	0.75	0.75	0.77	0.77	0.77	0.85	0.84	0.84
	item 2	0.73	0.72	0.72	0.73	0.73	0.74	0.78	0.78	0.78	0.85	0.85	0.86
	item 3	0.38	0.38	0.36	0.41	0.41	0.38	0.47	0.47	0.44	0.33	0.33	0.32
	item 4	0.46	0.46	0.45	0.50	0.50	0.48	0.58	0.58	0.56	0.32	0.32	0.30
	item 5	0.76	0.77	0.77	0.78	0.78	0.79	0.81	0.81	0.81	0.67	0.67	0.66
*Symbolization*	item 6	0.59	0.64	0.64	0.57	0.60	0.60	0.52	0.56	0.56	0.50	0.48	0.48
	item 7	0.72	0.75	0.75	0.79	0.82	0.82	0.75	0.78	0.78	0.71	0.79	0.79
	item 8	0.61	0.65	0.65	0.68	0.70	0.70	0.64	0.68	0.68	0.66	0.69	0.69
	item 9	0.66	0.56	0.56	0.67	0.58	0.58	0.65	0.53	0.53	0.57	0.41	0.41
	item 10	0.73	0.65	0.65	0.70	0.62	0.62	0.74	0.64	0.64	0.69	0.57	0.57
	cov(symbol; internalization)	0.58	0.58	0.58	0.47	0.48	0.49	0.54	0.58	0.59	0.54	0.60	0.61
	cov(item 9; item 10)		0.38	0.38		0.41	0.41		0.46	0.46		0.51	0.51
	cov(item 3; item 4)			0.16 *			0.24			0.26			0.22
RMSEA		0.06	0.03	0.02	0.09	0.07	0.06	0.10	0.07	0.06	0.11	0.08	0.07
RMSR		0.05	0.04	0.04	0.08	0.07	0.06	0.07	0.05	0.04	0.08	0.07	0.06
CFI		0.95	0.99	1.00	0.91	0.95	0.97	0.91	0.96	0.97	0.86	0.94	0.96
TLI		0.94	0.99	1.00	0.88	0.93	0.96	0.88	0.94	0.96	0.82	0.92	0.94
AIC		6664.84	6639.42	6635.34	16213.10	16131.47	16099.88	23252.11	23100.15	23045.72	21684.84	21479.38	21440.43
BIC		6775.81	6753.98	6753.47	16349.50	16272.28	16245.09	23400.95	23253.79	23204.16	21832.38	21631.67	21597.49

*Note*: All *b* coefficients were significant on *p*<0.001 if not stated otherwise as

* *p*<0.05 or

** *p*<0.01.

After verification of SMIS structure, sum scores of both subscales were correlated with MSC and MSI to establish validity evidence regarding relations to other constructs. Correlation coefficients are given in Table 4. Correlation between both SMIS subscales was positive and medium-sized (*r* = 0.41). Correlation of MSC with *Internalization* was positive but weak (*r* = 0.28), as in the case of *Symbolization* (*r* = 0.20). Correlations of MSI with *Internalization* were not significant (*r* = 0.05), while with *Symbolization* were positive and small (*r* = 0.26). Correlations of PPB were positive with both *Internalization* (*r* = 0.18) and *Symbolization* (*r* = 0.38).

**Table 4:** Correlation and partial correlation coefficients of SMIS with MSC, MSI and PPB across Studies 1, 2 and 3.

	Correlation coefficients					
	Study 1 (*n* = 529)		Study 2 (*n* = 602)		Study 3 (*n* = 899)	
	*Internalization*	*Symbolization*	*Internalization*	*Symbolization*	*Internalization*	*Symbolization*
*SMIS Symbolization*	0.41 ***	-	0.31 ***	-	0.41 ***	-
*Moral Self Concept*	0.28 ***	0.20 ***	0.35 ***	0.22 ***	0.44 ***	0.25 ***
*Moral Self-Image*	0.06	0.26 ***	0.05	0.18 ***	0.01	0.23 ***
*Past Prosocial Behaviors*	0.18 ***	0.38 ***	0.23 ***	0.37 ***	-	-
	Partial correlation coefficients					
	Study 1 (*n* = 529)		Study 2 (*n* = 602)		Study 3 (*n* = 899)	
	*Internalization*	*Symbolization*	*Internalization*	*Symbolization*	*Internalization*	*Symbolization*
*SMIS Symbolization*	0.35 ***	-	0.22 ***	-	0.36 ***	-
*Moral Self Concept*	0.25 ***	0.12 **	0.31 ***	0.14 ***	0.40 ***	0.01
*Moral Self-Image*	-0.03	0.18 ***	-0.05	0.11 **	-0.18 ***	0.22 ***
*Past Prosocial Behaviors*	0.15 **	0.35 ***	0.18 ***	0.33 ***	-	-

Note

* *p*<0.05

** *p*<0.01

*** *p*<0.001.

Partial correlations were also used to analyze validity while controlling for gender, age and other criterion constructs (MSC, MSI, PPB). Consistently with the correlation analysis, we observed a significant relationship of *Internalization* with MSC (*r*_*MSC*_ = 0.25, *p*<0.001), a nonsignificant relationship with MSI (*r*_*MSI*_ = -0.03, ns), a significant relationship with PPB (*r*_*PPB*_ = 0.15, *p* = 0.001) and significant relationships of *Symbolization* with those constructs (*r*_*MSC*_ = 0.12, *p* = 0.004, *r*_*MSI*_ = 0.18, *p*<0.001 and *r*_*PPB*_ = 0.35, *p*<0.001, respectively). The results are given in Table 4.

*Internalization* and *Symbolization* were predictive of declared donation of money for prevention of immoral behavior. The odds of donating money increased by 1.70 (95%CI 1.23, 2.33, *p* = 0.001) for each point increase in *Internalization* and by 1.85 (95%CI 1.42, 2.40, *p*<0.001) for point increase in *Symbolization*. The participants who declared donation of money scored significantly higher on *Internalization* (M_*Int*.*donation*_ = 4.19, *SD* = 0.57, *t*(527) = -5.63, *p*<0.001; Cohen’s *d* = -0.49) and *Symbolization* (M_*Sym*.*donation*_ = 3.20, *SD* = 0.69, *t*(527) = -6.68, *p*<0.001; Cohen’s *d* = -0.58) compared to those who did not want to donate any money (M_*Int*.*no-donation*_ = 3.88, *SD* = 0.69 and M_*Sym*.*no-donation*_ = 2.76 *SD* = 0.80, respectively).

In order to explore measurement invariance we tested previously constructed models 1–3 across gender using multigroup CFA in entire Study 1 sample (*n* = 529). Unconstrained models with significance tests of factor loadings between genders were presented in Table 5. Model 1 presented satisfactory fit (Model 1: RMSEA = 0.08, 90%CI 0.07–0.09, AIC = 13217.00, BIC = 13481.80, CFI = 0.93, TLI = 0.90), but was significantly improved by item 9 and 10 covariance (Model 2: LR test *χ2*(2) = 63.37, *p*<0.001, RMSEA = 0.06, 90%CI 0.04–0.07, AIC = 13157.63, BIC = 13430.97, CFI = 0.97, TLI = 0.95), and item 3 and 4 covariance (Model 3: LR test *χ2*(2) = 16.47, *p*<0.001, RMSEA = 0.05, 90%CI 0.03–0.07, AIC = 13145.16, BIC = 13427.04, CFI = 0.98, TLI = 0.97). Model 3 was chosen as configural model and was tested against the metric model, which did not fit the data worse (LR test *χ*^2^(8) = 14.44, *p* = 0.071, RMSEA = 0.05, 90%CI 0.03–0.07, AIC = 13143.60, BIC = 13391.32, CFI = 0.97, TLI = 0.96), thus supporting metric invariance. In the next step the metric model was compared to scalar model (with invariant loadings and intercepts) which presented similar fit (LR test *χ*^2^(8) = 7.18, *p* = 0.517, RMSEA = 0.05, 90%CI 0.03–0.06, AIC = 13134.78, BIC = 13348.33, CFI = 0.97, TLI = 0.97). Scalar model was compared to the error variance invariance model (invariant loadings, intercepts and residuals) which resulted in significantly poorer fit (LR test *χ*^2^(12) = 34.49, *p*< 0.001, RMSEA = 0.05, 90%CI 0.04–0.07, AIC = 13145.28, BIC = 13307.57, CFI = 0.96, TLI = 0.96) showing that measurement error differs across gender.

**Table 5:** Standardized coefficients of multigroup Confirmatory Factor Analysis (CFA) of SMIS items across gender in Studies 1–4.

		*Study 1 (n = 529)*								
		Model 1			Model 2			Model 3		
		*male b*	*female b*	*difference*	*male b*	*female b*	*difference*	*male b*	*female b*	*difference*
*Internalization*	item 1	0.65 ***	0.66 ***		0.65 ***	0.66 ***		0.65 ***	0.66 ***	
	item 2	0.70 ***	0.75 ***		0.70 ***	0.75 ***		0.69 ***	0.76 ***	
	item 3	0.31 **	0.45 ***		0.31 **	0.45 ***		0.27 *	0.43 ***	
	item 4	0.51 ***	0.53 ***		0.51 ***	0.53 ***		0.48 ***	0.51 ***	
	item 5	0.81 ***	0.74 ***		0.81 ***	0.75 ***		0.82 ***	0.75 ***	
*Symbolization*	item 6	0.69 ***	0.57 ***		0.70 ***	0.64 ***		0.70 ***	0.64 ***	
	item 7	0.84 ***	0.70 ***		0.86 ***	0.74 ***		0.86 ***	0.74 ***	
	item 8	0.59 ***	0.60 ***		0.59 ***	0.65 ***		0.59 ***	0.65 ***	
	item 9	0.41 ***	0.73 ***	**	0.37 ***	0.60 ***	**	0.37 ***	0.60 ***	**
	item 10	0.52 ***	0.80 ***	**	0.49 ***	0.69 ***	*	0.49 ***	0.69 ***	*
	cov(symbol; internalization)	0.40 ***	0.54 ***		0.38 **	0.57 ***		0.30 ***	0.14 ***	
	cov(item 9; item 10)				0.33 ***	0.44 ***		0.33 ***	0.44 ***	
	cov(item 3; item 4)							0.30 **	0.14 **	
RMSEA		0.08			0.06			0.05		
RMSR		0.05			0.04			0.03		
CFI		0.93			0.97			0.98		
TLI		0.90			0.95			0.97		
AIC		13217.00			13157.63			13145.16		
BIC		13481.80			13430.97			13427.04		
		*Study 2 (n = 602)*								
		Model 1			Model 2			Model 3		
		*male b*	*female b*	*difference*	*male b*	*female b*	*difference*	*male b*	*female b*	*difference*
*Internalization*	item 1	0.64 ***	0.81 ***		0.65 ***	0.81 ***		0.63 ***	0.81 ***	
	item 2	0.55 **	0.81 ***		0.55 ***	0.81 ***		0.54 ***	0.82 ***	
	item 3	0.21 ***	0.51 ***		0.21 *	0.51 ***		0.15 *	0.50 ***	*
	item 4	0.45 ***	0.51 ***		0.45 ***	0.51 ***		0.43 ***	0.49 ***	
	item 5	0.80 ***	0.79 ***		0.80 ***	0.79 ***		0.82 ***	0.79 ***	
*Symbolization*	item 6	0.61 ***	0.55 ***		0.62 ***	0.57 ***		0.62 ***	0.57 ***	
	item 7	0.74 ***	0.82 ***		0.78 ***	0.86 ***		0.78 ***	0.86 ***	
	item 8	0.67 ***	0.67 ***		0.69 ***	0.70 ***		0.68 ***	0.70 ***	
	item 9	0.67 ***	0.66 ***		0.59 ***	0.56 ***		0.60 ***	0.56 ***	
	item 10	0.60 ***	0.76 ***		0.51 ***	0.69 ***	*	0.51 ***	0.69 ***	*
	cov(symbol; internalization)	0.43 ***	0.46 ***		0.44 ***	0.47 ***		0.33 ***	0.17 ***	
	cov(item 9; item 10)				0.38 ***	0.44 ***		0.38 ***	0.44 ***	
	cov(item 3; item 4)							0.33 ***	0.17 **	*
RMSEA		0.10			0.07			0.06		
RMSR		0.08			0.07			0.06		
CFI		0.90			0.95			0.96		
TLI		0.87			0.93			0.95		
AIC		16164.17			16081.93			16051.71		
BIC		16436.99			16363.55			16342.13		
		*Study 3 (n = 899)*								
		Model 1			Model 2			Model 3		
		*male b*	*female b*	*difference*	*male b*	*female b*	*difference*	*male b*	*female b*	*difference*
*Internalization*	item 1	0.76 ***	0.76 ***		0.76 ***	0.76 ***		0.77 ***	0.76 ***	
	item 2	0.77 ***	0.77 ***		0.78 ***	0.77 ***		0.79 ***	0.77 ***	
	item 3	0.49 ***	0.43 ***		0.49 ***	0.43 ***		0.44 ***	0.41 ***	
	item 4	0.71 ***	0.50 ***		0.71 ***	0.49 ***		0.68 ***	0.47 ***	
	item 5	0.78 ***	0.80 ***		0.79 ***	0.80 ***		0.79 ***	0.80 ***	
*Symbolization*	item 6	0.55 ***	0.49 ***		0.58 ***	0.53 ***		0.58 ***	0.53 ***	
	item 7	0.70 ***	0.75 ***		0.76 ***	0.78 ***		0.76 ***	0.78 ***	
	item 8	0.61 ***	0.63 ***		0.68 ***	0.67 ***		0.68 ***	0.67 ***	
	item 9	0.71 ***	0.63 ***		0.55 ***	0.51 ***		0.55 ***	0.51 ***	
	item 10	0.83 ***	0.70 ***		0.72 ***	0.59 ***		0.72 ***	0.59 ***	
	cov(symbol; internalization)	0.51 ***	0.52 ***		0.57 ***	0.55 ***		0.30 ***	0.24 ***	
	cov(item 9; item 10)				0.48 ***	0.45 ***		0.48 ***	0.45 ***	
	cov(item 3; item 4)							0.30 ***	0.24 ***	
RMSEA		0.10			0.07			0.06		
RMSR		0.07			0.05			0.04		
CFI		0.90			0.95			0.97		
TLI		0.87			0.94			0.96		
AIC		23166.86			23020.73			22969.34		
BIC		23464.54			23328.01			23286.22		
		*Study 4 (n = 862)*								
		Model 1			Model 2			Model 3		
		*male b*	*female b*	*difference*	*male b*	*female b*	*difference*	*male b*	*female b*	*difference*
*Internalization*	item 1	0.87 ***	0.83 ***		0.86 ***	0.82 ***		0.86 ***	0.82 ***	
	item 2	0.81 ***	0.86 ***		0.82 ***	0.87 ***		0.82 ***	0.87 ***	
	item 3	0.34 ***	0.29 ***		0.34 ***	0.29 ***		0.32 ***	0.28 ***	
	item 4	0.34 ***	0.28 ***		0.34 ***	0.28 ***		0.33 ***	0.27 ***	
	item 5	0.64 ***	0.69 ***		0.64 ***	0.68 ***		0.64 ***	0.68 ***	
*Symbolization*	item 6	0.46 ***	0.52 ***		0.42 ***	0.50 ***		0.42 ***	0.50 ***	
	item 7	0.65 ***	0.73 ***		0.76 ***	0.81 ***		0.76 ***	0.81 ***	
	item 8	0.61 ***	0.65 ***		0.70 ***	0.65 ***		0.70 ***	0.65 ***	
	item 9	0.69 ***	0.45 ***	**	0.49 ***	0.31 ***	**	0.49 ***	0.31 ***	**
	item 10	0.77 ***	0.62 ***	*	0.60 ***	0.53 ***		0.60 ***	0.53 ***	
	cov(symbol; internalization)	0.40 ***	0.61 ***	**	0.52 ***	0.65 ***	*	0.52 ***	0.65 ***	*
	cov(item 9; item 10)				0.50 ***	0.53 ***		0.50 ***	0.53 ***	
	cov(item 3; item 4)							0.28 ***	0.15 **	
RMSEA		0.11			0.08			0.07		
RMSR		0.09			0.07			0.06		
CFI		0.85			0.93			0.95		
TLI		0.81			0.90			0.92		
AIC		21656.33			21461.14			21420.33		
BIC		21951.40			21765.73			21734.44		

Note

* p<0.05;

** p<0.01;

*** p<0.001.

### Study 2

Study 2 was conducted on the sample of 602 participants to confirm the factor structure on a more balanced sample. The descriptive statistics of the sample are given in Table 1. In order to assess dimensionality, PA and EGA were conducted leading to detection of two main dimensions consisting of the same items as in Study 1 (S1 and S2 Figs). Next, CFA of SMIS items was conducted resulting in poorly fitting simple structure model (Model 1: RMSEA = 0.09, 90%CI: 0.08–0.11, AIC = 16213.10, BIC = 16349.50, CFI = 0.91, TLI = 0.88). Following item contents and MI we added covariance of items 9 and 10, which significantly improved fit (Model 2: LR test: *χ*^2^(1) = 83.62 *p*<0.001, RMSEA = 0.07, 90%CI 0.06–0.08, AIC = 16131.47, BIC = 16272.28, CFI = 0.95, TLI = 0.93). Covariance of items 3 and 4 also was introduced resulting in better fitting model (LR test *χ*^2^(1) = 33.59 *p*<0.001, Model 3: RMSEA = 0.06, 90%CI 0.04–0.07, AIC = 16099.88, BIC = 16245.09, CFI = 0.97, TLI = 0.96). CFA models are given in Table 3 in Study 2 section. Reliability assessed using the alpha coefficient was *α*_*Internalization*_ = 0.76 and *α*_*Symbolization*_ = 0.81, *α*_*MSCS*_ = 0.80, *α*_*MSIS*_ = 0.77 and *α*_*RDM*_ = 0.58.

Validity evidence were provided by correlations and partial correlations of *Internalization* and *Symbolization* with MSC, MSI and PPB. *Internalization* correlated with MSC (*r* = 0.35) and PPB (*r* = 0.23) but not with MSI (*r* = 0.05). *Symbolization* correlated with MSC (*r* = 0.22), MSI (*r* = 0.18) and PPB (*r* = 0.37). Partial correlations of *Internalization* with MSC (*r*_*MSC*_ = 0.31, *p*<0.001) and PPB (*r*_*PPB*_ = 0.18, *p*<0.001) were significant but not with MSI (*r*_*MSI*_ = -0.05, *p* = 0.275), while *Symbolization* correlated with all of them, MSC (*r*_*MSC*_ = 0.14, *p*<0.001), MSI (*r*_*MSI*_ = 0.11, *p* = 0.006) and PPB (*r*_*MSI*_ = 0.33, *p*<0.001). The results are given in Table 4. The odds of money donation increased by 1.33 (95%CI 1.02–1.75, *p* = 0.037) for each point increase in *Internalization* and by 1.28 (95%CI 1.03–1.58, *p* = 0.025) for point increase in *Symbolization*. The participants who donated money for prevention of immoral behavior scored significantly higher on *Internalization* (M_*Int*.*donation*_ = 4.16, *SD* = 0.67, *t*(600) = -2.93, *p* = 0.004; Cohen’s *d* = -0.26) as in the case of *Symbolization* (M_*Sym*.*donation*_ = 2.94, *SD* = 0.91, *t*(600) = -3.07, *p* = 0.002; Cohen’s *d* = -0.28) compared to those who did not express the will to donate money (M_*Int*.*no-donation*_ = 3.97, *SD* = 0.74 and M_*Sym*.*no-donation*_ = 2.69 *SD* = 0.87, respectively). CFA models were tested across gender in multigroup CFA (Table 5). Poorly fitting initial model (Model 1: RMSEA = 0.10, 90%CI 0.09–0.11, AIC = 16164.17, BIC = 16436.99, CFI = 0.90, TLI = 0.87) has been significantly improved by adding covariance between items 9 and 10 (Model 2: LR test *χ*^2^(2) = 86.24, *p*<0.001, RMSEA = 0.07, 90%CI 0.06–0.09, AIC = 16081.93, BIC = 16363.55, CFI = 0.95, TLI = 0.93), and items 3 and 4 resulting in excellent fit (Model 3: LR test *χ*^2^(2) = 34.22, *p*<0.001, RMSEA = 0.06, 90%CI 0.05–0.08, AIC = 16051.71, BIC = 16342.13, CFI = 0.96, TLI = 0.95). Model 3 was chosen as configural model and was tested against the metric model, which did fit the data better (LR test *χ*^2^(8) = 21.82, *p* = 0.005, RMSEA = 0.06, 90%CI 0.05–0.08, AIC = 16057.53, BIC = 16312.74, CFI = 0.96, TLI = 0.94) leading to conclusion that factor loadings differ across gender.

### Study 3

Study 3 sample of 899 participants was used to attempt to replicate SMIS factor structure using CFA on an older sample. The descriptive statistics of the sample are given in Table 1. Dimensionality assessment using PA and EGA lead to detection of two major factors (S1 and S2 Figs). The fit of the simple structure CFA model was poor (Model 1: RMSEA = 0.10, 90%CI 0.09–0.11, AIC = 23252.11, BIC = 23400.95, CFI = 0.91, TLI = 0.88). Covariance of items 9 and 10 was added, which considerably improved the fit (Model 2: LR test *χ*^2^(1) = 153.96, *p*<0.001, RMSEA = 0.07, 90%CI 0.06–0.08, AIC = 23100.15, BIC = 23253.79, CFI = 0.96, TLI = 0.94), and the covariance between items 3 and 4, which resulted in good fit (Model 3: LR test *χ*^2^(1) = 56.43, *p*<0.001, RMSEA = 0.06, 90%CI 0.04–0.07, AIC = 23045.72, BIC = 23204.16, CFI = 0.97, TLI = 0 .96; Table 3 in Study 3 section). The alpha coefficient for each measure was *α*_*Internalization*_ = 0.81 and *α*_*Symbolization*_ = 0.79, *α*_*MSCS*_ = 0.80 and *α*_*MSIS*_ = 0.82.

*Internalization* correlated with MSC (*r* = 0.44) but not with MSI (*r* = 0.01), while *Symbolization* correlated with both measures (*r* = 0.25 and *r* = 0.23, respectively). Partial correlation of *Internalization* with MSC was positive (*r*_*MSC*_ = 0.40, *p*<0.001), and negative with MSI (*r*_*MSI*_ = -0.18, *p*<0.001). The results are given in Table 4.

The odds of money donation increased by 2.00 (95%CI 1.14–3.49, *p* = 0.015) for point increase in *Internalization* but not in *Symbolization* (OR = 1.33, 95%CI 0.73–2.42, *p* = 0.354). The mean score of *Internalization* and *Symbolization* in participants who declared money donation was higher (M_*Int*.*donation*_ = 4.16, *SD* = 0.69, *t*(35.34) = -2.92, *p* = 0.006; Cohen’s *d* = -0.70) but not on *Symbolization* (M_*Sym*.*donation*_ = 2.99, *SD* = 0.70, *t*(35.13) = -1.93, *ns*; Cohen’s *d* = -0.47) compared to those who did not donate money (M_*Int*.*no-donation*_ = 3.65, *SD* = 0.90 and M_*Sym*.*no-donation*_ = 2.64, *SD* = 0.94, respectively).

Measurement invariance across gender was tested in multigroup CFA (Table 5). Initial model presented poor fit (Model 1: RMSEA = 0.10, 90%CI 0.09–0.11, AIC = 23166.86, BIC = 23464.54, CFI = 0.90, TLI = 0.87), which was significantly improved by adding covariance of items 9 and 10 (Model 2: LR test *χ*^2^(2) = 150.13, *p*<0.001, RMSEA = 0.07, 90%CI 0.06–0.08, AIC = 21461.14, BIC = 21765.73, CFI = 0.95, TLI = 0.94) as well as items 3 and 4 (Model 3: LR test *χ*^2^(2) = 55.39, *p*<0.001, RMSEA = 0.06, 90%CI 0.05–0.07, AIC = 22969.34, BIC = 23286.22, CFI = 0.97, TLI = 0.96). The metric model did not fit the data worse than the configural model (Model 3) providing evidence for metric invariance (LR test *χ*^2^(8) = 9.73, *p* = 0.285, RMSEA = 0.05, 90%CI 0.04–0.06, AIC = 22963.07, BIC = 23241.54, CFI = 0.97, TLI = 0.96). The scalar model presented similar fit as the metric model (LR test *χ*^2^(8) = 7.75, *p* = 0.458, RMSEA = 0.05, 90%CI 0.04–0.06, AIC = 22954.82, BIC = 23194.88, CFI = 0.97, TLI = 0.97), and then was compared to the error variance invariance model which resulted in significantly poorer fit (LR test *χ*^2^(12) = 45.16, *p*< 0.001, RMSEA = 0.06, 90%CI 0.05–0.06, AIC = 22975.97, BIC = 23158.42, CFI = 0.96, TLI = 0.96) indicating different measurement error across gender.

### Study 4

A sample of 862 participants with balanced gender (50% women) and higher age was used to replicate the factor structure with CFA. Dimensionality was assessed based on eigenvalues PA plot and EGA graph (S1 and S2 Figs).

The fit of the simple structure CFA model was poor (Model 1: RMSEA = 0.11, 90%CI 0.10–0.12, AIC = 21684.84, BIC = 21832.38, CFI = 0.86, TLI = 0.82). Covariance of items 9 and 10 was added, which significantly improved the fit (Model 2: LR test *χ*^2^(1) = 207.46, *p*<0.001, RMSEA = 0.08, 90%IC 0.07–0.09, AIC = 21479.38, BIC = 21631.67, CFI = 0.94, TLI = 0.92), and the covariance between items 3 and 4 was also added (Model 3: LR test *χ*^2^(1) = 40.95, *p*<0.001, RMSEA = 0.07, 90%CI 0.06–0.08, AIC = 21440.43, BIC = 21597.49, CFI = 0.96, TLI = 0.94; Table 3 in Study 4 section). The alpha coefficient reliability was *α*_*Internalization*_ = 0.71 and *α*_*Symbolization*_ = 0.76.

The odds of donating money for public health increased by 1.55 (95%CI 1.13–2.14, *p* = 0.007) for unit increase in *Internalization* and by 1.78 for unit increase in *Symbolization* (OR = 1.78, 95%CI 1.32–2.39, *p*<0.001). The mean score of *Internalization* and *Symbolization* in participants who declared money donation was higher (M_*Int*.*donation*_ = 4.14, *SD* = 0.62, *t*(122.09) = -3.60, *p* = 0.001; Cohen’s *d* = -0.43 and M_*Sym*.*donation*_ = 3.09, *SD* = 0.72, *t*(124.37) = -4.43, *p*<0.001; Cohen’s *d* = -0.51) compared to the participants who did not declared donation (M_*Int*.*no-donation*_ = 3.87, *SD* = 0.75 and M_*Sym*.*no-donation*_ = 2.71, *SD* = 0.82, respectively).

Measurement invariance across gender was tested in multigroup CFA (Table 5). Poor fitting initial model (Model 1: RMSEA = 0.11, 90%CI 0.10–0.12, AIC = 21656.33, BIC = 21951.40, CFI = 0.85, TLI = 0.81) was improved by covariances between items 9 and 10 (Model 2: LR test *χ*^2^(2) = 199.19, *p*<0.001, RMSEA = 0.08, 90%CI 0.07–0.09, AIC = 21461.14, BIC = 21765.73, CFI = 0.93, TLI = 0.90) and items 3 and 4, which resulted in good fit (Model 3: LR test *χ*^2^(2) = 44.81, *p*<0.001, RMSEA = 0.07, 90%CI 0.06–0.08, AIC = 21420.33, BIC = 21734.44, CFI = 0.95, TLI = 0.92). The metric model did not fit the data worse than the configural model (LR test *χ*^2^(8) = 10.97, *p* = 0.204, RMSEA = 0.06, 90%CI 0.06–0.08, AIC = 21415.29, BIC = 21691.33, CFI = 0.95, TLI = 0.93), thus supporting metric invariance. In the next step the metric model was compared to scalar model which presented similar fit (LR test *χ*^2^(8) = 7.64, *p* = 0.469, RMSEA = 0.06, 90%CI 0.05–0.07, AIC = 21406.94, BIC = 21644.90, CFI = 0.95, TLI = 0.94). Error variance invariance model compared to the scalar model presented significantly poorer fit (LR test *χ*^2^(12) = 22.52, *p* = 0.032, RMSEA = 0.06, 90%CI 0.05–0.07, AIC = 21405.46, BIC = 21586.31, CFI = 0.94, TLI = 0.94) suggesting that measurement error differs across gender.

Additionally, normalization for men and women was created based on Study 4 (see: S1 Table).

## General discussion

Replicability of psychometric characteristics and re-establishing validity are essential for adequate psychological measurement. Our research has shown that SMIS is a psychometrically stable measure of moral identity and appears to be a valid measure in the Polish culture.

Four studies were conducted to verify adequacy of the validation of SMIS in the Polish population. Study 1 (EFA) replicated factors originally obtained by Aquino & Reed [11], which were cross-validated in Study 1 (CFA), achieving very good fit both with and without the covariance of items 9 and 10. Studies 2 and 3 replicated the findings of Study 1 with a satisfactory model fit after the covariance of items 9 and 10 was introduced to the models. Model 3 improved the already good fit with the covariance of items 3 and 4. To address the problem of replicating studies with the same shortcomings, Study 4 with a more representative sample with balanced gender, education, and age was used. Satisfactory model fit was achieved with the covariance of items 9 and 10 and further improved with the covariance of items 3 and 4. These results proved the replicability of the overall structure of SMIS and consequently indicated closer relation of items considering the communication of moral values by external actions (items 9 & 10), and the rejection of moral values (items 3 & 4; also reverse coding might have caused the correlation of the error terms). No bifactor solution was sought since both covariances were identified within factors, which is consistent with no cross-loadings structure, and adequate fit was achieved with two factors, thus non-substantial factor was considered unwarranted [40]. We do not know the magnitude of analogous covariances in the original study by Aquino & Reed [11] neither the replicability of their model fit on different samples, thus it is hard to determine whether the source of those covariances might lie in translation process, cultural or populational differences, or true dimensionality of SMIS.

The validity of SMIS was assessed as evidence for content validity throughout stages of translation procedure, evidence related to internal structure in form of factorial structure and measurement invariance assessment, and evidence of relations to other constructs. In Studies 1–4 we established convergent validity evidence with morality-based constructs, MSC [24], and MSI [25], as well as PPB [26] and RDM which might be considered test-criterion. MSC correlated with both *Internalization* and *Symbolization*, which is consistent with earlier research (Study 4) [11]. MSI correlated with *Symbolization* but not with *Internalization*, which is in line with previous studies [25]. We also showed that both *Internalization* and *Symbolization* subscales were related to PPB, which is consistent with the relation between the self-importance of moral identity and self-reported volunteerism (Study 5) [11], and moral behavior [18]. Finally, the self-importance of moral identity was also positively related to RDM, which confirmed past results on actual donation behaviors (Study 6) [11, 18]. The pattern of correlations of the self-importance of moral identity and other moral measures was consistent and stable across all studies even while controlling for other constructs.

Measurement invariance across gender was tested in each study supporting strong invariance (invariant loadings and intercepts) in Studies 1, 3 and 4 but not Study 2. Slight differences in item loadings were detected in items 9 and 10, especially in Study 4 which was the most age and gender balanced. These items indicate communication of moral characteristics which might be related to gender in Polish population. It is consistent with overall lower individualism and more pronounced gender roles in Polish society. It is likely that public manifestation of one’s values will be constricted by societal norms to some extent in any culture. Although gender measurement invariance in SMIS was inspected before [40, 41], to our best knowledge our study is the first to detect any differences related to gender.

The main limitation of the research is its cross-sectional character which does not allow for experimental manipulation of moral identity or the analysis of morally specific populations which would be the most reliable validity assessments, instead of correlational and declarative validity evidence. The main strengths of the research are (1) use of reliable translation method; (2) consistent replications;(3) the use of the representative sample in Study 4 which facilitates generalizability of the findings; (4) sample size providing sufficient statistical power in each study; (5) assessment of various validity constructs; (6) assessment of gender measurement invariance and (7) developing norms for men and women.

## Conclusions

Four consecutive studies were conducted to evaluate psychometric properties and validity of the Polish version of SMIS. Stability of the factorial structure and relation to other moral constructs were proven to be consistent with the original research of the English version of SMIS. Gender differences were detected in overall scores on SMIS as well as factor loadings on items 9 (communicating by membership in organizations) and 10 (communicating by activities). The analysis revealed that women tended to have higher scores compared to men.

## Supporting information

S1 Fig Scree plot for parallel analysis in Study 1–4. In order to assess dimensionality in Study 1, Parallel Analysis (with 100 replications) was conducted on subsample A, Scree plot is presented in Fig 1. Parallel Analysis was also used to analyze dimensionality in Study 2, Study 3 and Study 4. *Note*: Due to some concerns that results may be biased because of split sample strategy used in Study 1, PA was also conducted on the whole Study 1 sample (*N* = 529) and the results were virtually identical.
(TIF)

S2 Fig Exploratory Graph Analysis (EGA) for Study 1 subsample A (A), Study 2 (B) Study 3 (C), and Study 4 (D). Further analysis of dimensionality using EGA resulted in detection of two main dimensions with considerable relations between items 9–10, 3–4 and 1-2-5 (Fig 2).
(TIF)

S1 File Polish version of the Self-Importance of Moral Identity Scale (Paruzel-Czachura & Blukacz, 2021). Please cite this paper as a source of the Polish version.
(DOCX)

S1 Table Polish normalization of SMIS. Percentile distribution of the SMIS Internalization and Symbolization scores by gender in Study 4.
(DOCX)
