# Supplementary figures and images for: How relevant for you is to be a moral person? Polish validation of the Self-Importance of Moral Identity Scale
Source: PLoS One. 2021 Aug 3;16(8):e0255386. doi: 10.1371/journal.pone.0255386 (PMC8330904; doi:10.1371/journal.pone.0255386)

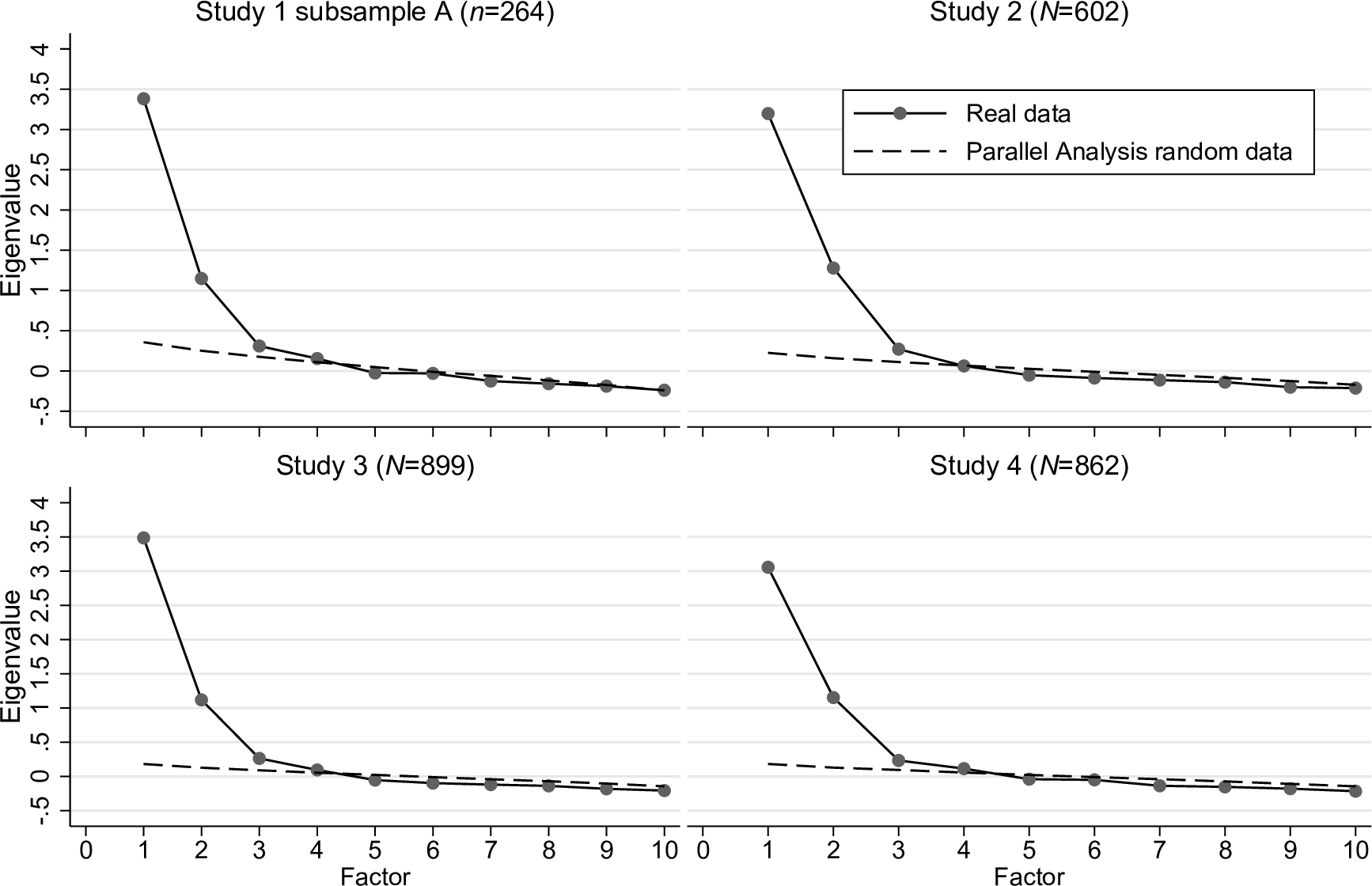

Supplement: S1 Fig — In order to assess dimensionality in Study 1, Parallel Analysis (with 100 replications) was conducted on subsample A, Scree plot is presented in Fig 1. Parallel Analysis was also used to analyze dimensionality in Study 2, Study 3 and Study 4. Note: Due to some concerns that results may be biased because of split sample strategy used in Study 1, PA was also conducted on the whole Study 1 sample (N = 529) and the results were virtually identical. (TIF) [file pone.0255386.s001.tif]

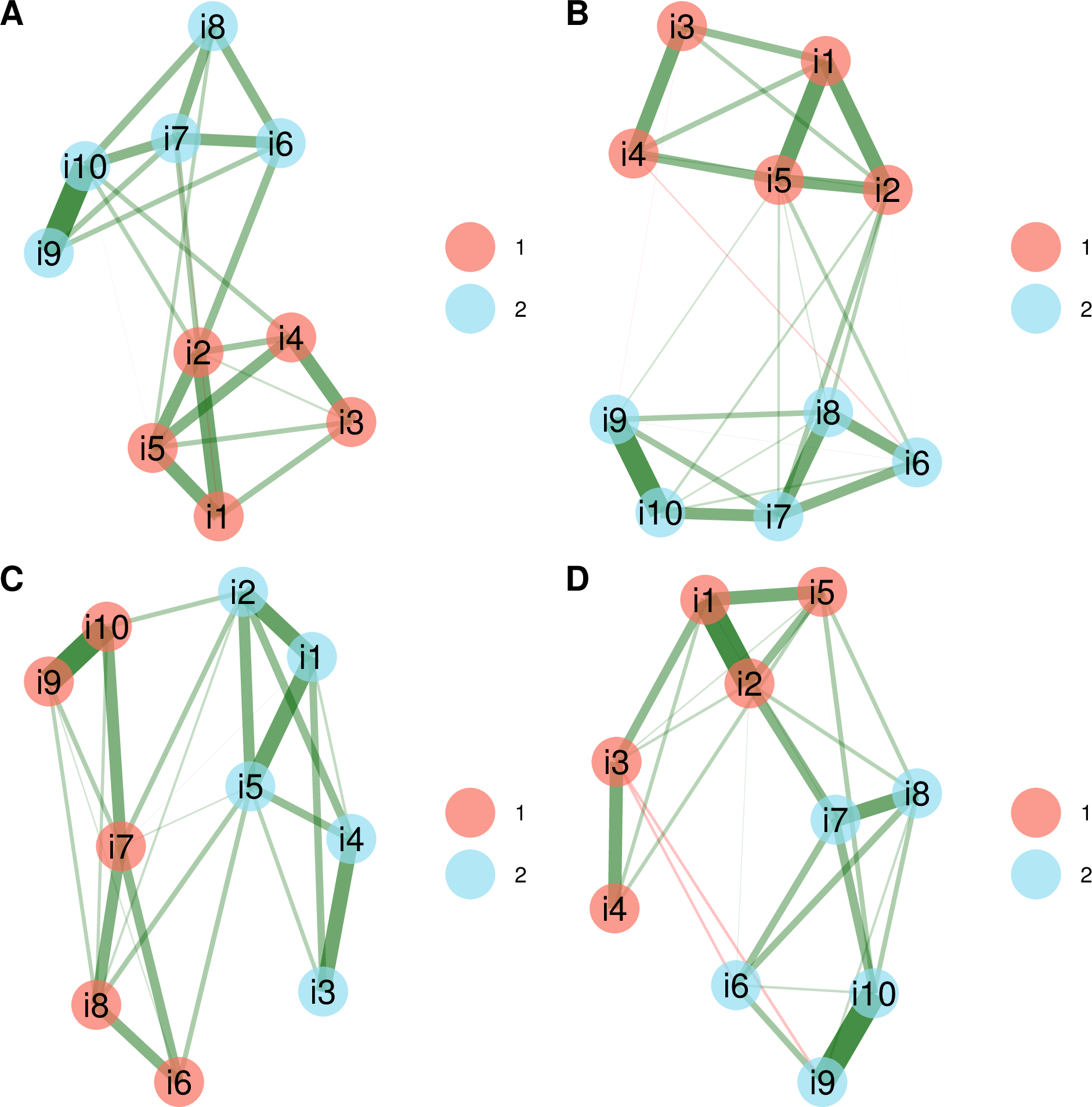

Supplement: S2 Fig — Exploratory Graph Analysis (EGA) for Study 1 subsample A (A), Study 2 (B) Study 3 (C), and Study 4 (D). Further analysis of dimensionality using EGA resulted in detection of two main dimensions with considerable relations between items 9–10, 3–4 and 1-2-5 (Fig 2). (TIF) [file pone.0255386.s002.tif]
