# Supplementary material for: How relevant for you is to be a moral person? Polish validation of the Self-Importance of Moral Identity Scale
Source: PLoS One. 2021 Aug 3;16(8):e0255386. doi: 10.1371/journal.pone.0255386 (PMC8330904; doi:10.1371/journal.pone.0255386)
Supplement: S1 File — Please cite this paper as a source of the Polish version. (DOCX) [file pone.0255386.s003.docx]

**Skala Znaczenia Osobistej Tożsamości Moralnej**

**(Polska walidacja: Mariola Paruzel-Czachura & Mateusz Blukacz, 2021)**

Poniżej wymieniono niektóre cechy, którymi można opisać człowieka:

*opiekuńczy, współczujący, sprawiedliwy, przyjazny, hojny, pomocny, pracowity, uczciwy i życzliwy.*

Osobą o tym zestawie cech możesz być Ty lub ktoś inny. Przez chwilę wyobraź sobie osobę
o tych cechach. Wyobraź sobie co taka osoba mogłaby myśleć, czuć i robić. Jeśli masz już wyraźny obraz takiej osoby, odpowiedz na poniższe pytania na skali:

*1 - Zdecydowanie się nie zgadzam*

*2 - Nie zgadzam się*

*3 - Ani się nie zgadzam ani się zgadzam*

*4 - Zgadzam się*

*5 - Zdecydowanie się zgadzam*

1. Czułbym/abym się dobrze będąc osobą o takich cechach.

2. Bycie osobą posiadającą te cechy jest ważną częścią tego, kim jestem.

3. Wstydziłbym/abym się być osobą posiadającą te cechy. (R)

4. Posiadanie tych cech nie jest tak naprawdę dla mnie ważne. (R)

5. Mocno pragnę posiadać takie cechy.

6. Często noszę ubrania, które wskazują na posiadanie przeze mnie powyższych cech.

7. Różne czynności, które robię w wolnym czasie (np. hobby) wyraźnie pokazują,
że jestem osobą o takich cechach.

8. Rodzaje książek i artykułów, jakie czytam, wskazują na to, że takie cechy posiadam.

9. Fakt, że posiadam takie cechy, jest widoczny dla innych w tym, że jestem członkiem pewnych organizacji.

10. Jestem aktywnie zaangażowany w czynności, które pokazują innym, że posiadam takie cechy.

*Skala internalizacji*: 1, 2, 3, 4, 5

*Skala symbolizacji*: 6, 7, 8, 9, 10

R – item odwrócony
