# Supplementary material for: How relevant for you is to be a moral person? Polish validation of the Self-Importance of Moral Identity Scale
Source: PLoS One. 2021 Aug 3;16(8):e0255386. doi: 10.1371/journal.pone.0255386 (PMC8330904; doi:10.1371/journal.pone.0255386)
Supplement: S1 Table — Percentile distribution of the SMIS Internalization and Symbolization scores by gender in Study 4. (DOCX) [file pone.0255386.s004.docx]

| **S1 Table. Polish Normalization of SMIS.** Percentile distribution of the SMIS Internalization and Symbolization scores by gender in Study 4. | | | | | |
| --- | --- | --- | --- | --- | --- |
|  | Male (*n* = 435) | |  | Female (*n* = 427) | |
| Percentile | *Internalization* | *Symbolization* |  | *Internalization* | *Symbolization* |
| 1 | 2.4 | 1.0 |  | 2.4 | 1.4 |
| 2 | 2.8 | 1.2 |  | 2.8 | 1.6 |
| 3 | 3.0 | 1.4 |  | 3.0 | 1.6 |
| 4 | 3.0 | 1.6 |  | 3.0 | 1.8 |
| 5 | 3.0 | 1.6 |  | 3.2 | 2.0 |
| 6 | 3.0 | 1.8 |  | 3.2 | 2.0 |
| 7 | 3.0 | 1.8 |  | 3.2 | 2.0 |
| 8 | 3.2 | 1.8 |  | 3.2 | 2.2 |
| 9 | 3.2 | 2.0 |  | 3.4 | 2.2 |
| 10 | 3.2 | 2.0 |  | 3.4 | 2.2 |
| 11 | 3.2 | 2.0 |  | 3.4 | 2.4 |
| 12 | 3.2 | 2.0 |  | 3.4 | 2.4 |
| 13 | 3.2 | 2.0 |  | 3.4 | 2.4 |
| 14 | 3.2 | 2.2 |  | 3.4 | 2.4 |
| 15 | 3.4 | 2.2 |  | 3.6 | 2.6 |
| 16 | 3.4 | 2.2 |  | 3.6 | 2.6 |
| 17 | 3.4 | 2.2 |  | 3.6 | 2.6 |
| 18 | 3.4 | 2.2 |  | 3.6 | 2.6 |
| 19 | 3.4 | 2.2 |  | 3.6 | 2.6 |
| 20 | 3.4 | 2.2 |  | 3.8 | 2.6 |
| 21 | 3.4 | 2.4 |  | 3.8 | 2.6 |
| 22 | 3.4 | 2.4 |  | 3.8 | 2.6 |
| 23 | 3.6 | 2.4 |  | 3.8 | 2.6 |
| 24 | 3.6 | 2.4 |  | 3.8 | 2.8 |
| 25 | 3.6 | 2.4 |  | 3.8 | 2.8 |
| 26 | 3.6 | 2.4 |  | 3.8 | 2.8 |
| 27 | 3.6 | 2.6 |  | 3.8 | 2.8 |
| 28 | 3.6 | 2.6 |  | 3.8 | 2.8 |
| 29 | 3.6 | 2.6 |  | 3.8 | 2.8 |
| 30 | 3.6 | 2.6 |  | 4.0 | 3.0 |
| 31 | 3.6 | 2.6 |  | 4.0 | 3.0 |
| 32 | 3.6 | 2.6 |  | 4.0 | 3.0 |
| 33 | 3.6 | 2.6 |  | 4.0 | 3.0 |
| 34 | 3.8 | 2.6 |  | 4.0 | 3.0 |
| 35 | 3.8 | 2.6 |  | 4.0 | 3.0 |
| 36 | 3.8 | 2.6 |  | 4.0 | 3.0 |
| 37 | 3.8 | 2.6 |  | 4.0 | 3.0 |
| 38 | 3.8 | 2.8 |  | 4.0 | 3.0 |
| 39 | 3.8 | 2.8 |  | 4.2 | 3.0 |
| 40 | 3.8 | 2.8 |  | 4.2 | 3.0 |
| 41 | 3.8 | 2.8 |  | 4.2 | 3.0 |
| 42 | 3.8 | 2.8 |  | 4.2 | 3.0 |
| 43 | 3.8 | 2.8 |  | 4.2 | 3.0 |
| 44 | 3.8 | 2.8 |  | 4.2 | 3.0 |
| 45 | 4.0 | 2.8 |  | 4.2 | 3.0 |
| 46 | 4.0 | 2.8 |  | 4.2 | 3.0 |
| 47 | 4.0 | 2.8 |  | 4.2 | 3.2 |
| 48 | 4.0 | 3.0 |  | 4.2 | 3.2 |
| 49 | 4.0 | 3.0 |  | 4.2 | 3.2 |
| 50 | 4.0 | 3.0 |  | 4.2 | 3.2 |
| 51 | 4.0 | 3.0 |  | 4.2 | 3.2 |
| 52 | 4.0 | 3.0 |  | 4.2 | 3.2 |
| 53 | 4.0 | 3.0 |  | 4.2 | 3.2 |
| 54 | 4.0 | 3.0 |  | 4.4 | 3.2 |
| 55 | 4.2 | 3.0 |  | 4.4 | 3.2 |
| 56 | 4.2 | 3.0 |  | 4.4 | 3.2 |
| 57 | 4.2 | 3.0 |  | 4.4 | 3.2 |
| 58 | 4.2 | 3.0 |  | 4.4 | 3.4 |
| 59 | 4.2 | 3.0 |  | 4.4 | 3.4 |
| 60 | 4.2 | 3.0 |  | 4.4 | 3.4 |
| 61 | 4.2 | 3.0 |  | 4.4 | 3.4 |
| 62 | 4.2 | 3.0 |  | 4.4 | 3.4 |
| 63 | 4.2 | 3.2 |  | 4.6 | 3.4 |
| 64 | 4.2 | 3.2 |  | 4.6 | 3.4 |
| 65 | 4.2 | 3.2 |  | 4.6 | 3.4 |
| 66 | 4.2 | 3.2 |  | 4.6 | 3.4 |
| 67 | 4.4 | 3.2 |  | 4.6 | 3.4 |
| 68 | 4.4 | 3.2 |  | 4.6 | 3.4 |
| 69 | 4.4 | 3.2 |  | 4.6 | 3.4 |
| 70 | 4.4 | 3.2 |  | 4.6 | 3.4 |
| 71 | 4.4 | 3.2 |  | 4.6 | 3.6 |
| 72 | 4.4 | 3.4 |  | 4.8 | 3.6 |
| 73 | 4.4 | 3.4 |  | 4.8 | 3.6 |
| 74 | 4.4 | 3.4 |  | 4.8 | 3.6 |
| 75 | 4.4 | 3.4 |  | 4.8 | 3.6 |
| 76 | 4.6 | 3.4 |  | 4.8 | 3.6 |
| 77 | 4.6 | 3.4 |  | 4.8 | 3.6 |
| 78 | 4.6 | 3.4 |  | 4.8 | 3.6 |
| 79 | 4.6 | 3.6 |  | 4.8 | 3.6 |
| 80 | 4.6 | 3.6 |  | 4.8 | 3.8 |
| 81 | 4.6 | 3.6 |  | 4.8 | 3.8 |
| 82 | 4.6 | 3.6 |  | 5.0 | 3.8 |
| 83 | 4.6 | 3.6 |  | 5.0 | 3.8 |
| 84 | 4.8 | 3.8 |  | 5.0 | 3.8 |
| 85 | 4.8 | 3.8 |  | 5.0 | 3.8 |
| 86 | 4.8 | 3.8 |  | 5.0 | 4.0 |
| 87 | 4.8 | 3.8 |  | 5.0 | 4.0 |
| 88 | 4.8 | 3.8 |  | 5.0 | 4.0 |
| 89 | 4.8 | 3.8 |  | 5.0 | 4.0 |
| 90 | 5.0 | 4.0 |  | 5.0 | 4.0 |
| 91 | 5.0 | 4.0 |  | 5.0 | 4.0 |
| 92 | 5.0 | 4.0 |  | 5.0 | 4.2 |
| 93 | 5.0 | 4.0 |  | 5.0 | 4.2 |
| 94 | 5.0 | 4.0 |  | 5.0 | 4.2 |
| 95 | 5.0 | 4.0 |  | 5.0 | 4.4 |
| 96 | 5.0 | 4.0 |  | 5.0 | 4.6 |
| 97 | 5.0 | 4.2 |  | 5.0 | 4.6 |
| 98 | 5.0 | 4.4 |  | 5.0 | 4.8 |
| 99 | 5.0 | 4.6 |  | 5.0 | 5.0 |
